# Intracellular Survival and Persistence of *Chlamydia muridarum* Is Determined by Macrophage Polarization

**DOI:** 10.1371/journal.pone.0069421

**Published:** 2013-08-14

**Authors:** Eric Gracey, Aifeng Lin, Ali Akram, Basil Chiu, Robert D. Inman

**Affiliations:** 1 Department of Immunology, University of Toronto, Toronto, Ontario, Canada; 2 University Health Network, Toronto, Ontario, Canada; Duke University Medical Center, United States of America

## Abstract

Macrophages can display a number of distinct phenotypes, known collectively as polarized macrophages. The best defined of these phenotypes are the classically-activated, interferon gamma (IFNγ)/LPS induced (M1) and alternatively-activated, IL-4 induced (M2) macrophages. The goal of this study is to characterize macrophage- *Chlamydia* interactions in the context of macrophage polarization. Here we use *Chlamydia muridarum* and murine bone-marrow derived macrophages to show *Chlamydia* does not induce M2 polarization in macrophages as a survival strategy. Unexpectedly, the infection of macrophages was silent with no upregulation of M1 macrophage-associated genes. We further demonstrate that macrophages polarized prior to infection have a differential capacity to control *Chlamydia* . M1 macrophages harbor up to 40-fold lower inclusion forming units (IFU) than non-polarized or M2 polarized macrophages. Gene expression analysis showed an increase in *16sRNA* in M2 macrophages with no change in M1 macrophages. Suppressed *Chlamydia* growth in M1 macrophages correlated with the induction of a bacterial gene expression profile typical of persistence as evident by increased *Euo* expression and decreased *Omp1* and *Tal* expression. Observations of permissive *Chlamydia* growth in non-polarized and M2 macrophages and persistence in M1 macrophages were supported through electron microscopy. This work supports the importance of IFNγ in the innate immune response to *Chlamydia* . However, demonstration that the M1 macrophages, despite an antimicrobial signature, fail to eliminate intracellular *Chlamydia* supports the notion that host–pathogen co-evolution has yielded a pathogen that can evade cellular defenses against this pathogen, and persist for prolonged periods of time in the host.

## Introduction

The Chlamydiaceae are a family of pathogens which have evolved closely with their hosts for millennia [1]. *Chlamydia trachomatis* is the most common cause of infectious blindness and the most common sexually transmitted bacterial infection in humans [2]. The murine pathogen *Chlamydia muridarum*, a close relative of *C. trachomatis* [3], is commonly employed for animal models of human *Chlamydia* infections.

Understanding of the pathogenesis of these organisms has been limited by their obligate intracellular nature and complex biphasic lifecycle. Extracellular elementary bodies (EB) are infectious but non-replicative, whereas intracellular reticulate bodies (RB) are non-infectious but replicative [4]. The RB are found in bacteria-modified vesicles, or inclusions. A third, stress-induced stage in the organism’s life cycle has been identified, persistence, in which *Chlamydia* grow but do not divide, resulting in enlarged aberrantly shaped RB. *In vitro* persistence is induced by interferon gamma (IFNγ) and antibiotics, and has been well characterized in epithelial cells and fibroblasts. Spontaneous persistence in mononuclear phagocytes has also been observed [5]. Traditionally persistence was defined as the circumstance in which *Chlamydia* could be directly detected in infected cells through microscopy with a reduced ability to culture. Recent work has characterized persistence on the molecular level with gene and protein expression profiles [5].

Despite its induction of persistence *in vitro*, IFNγ has been shown unambiguously to be essential for the control of *Chlamydia* , particularly for the host innate immune response [6,7]. At the point of entry, *Chlamydia* infect epithelial cells which are permissive to their growth. This rapidly elicits an innate immune response consisting of mononuclear cells, polymorphonuclear phagocytes and innate lymphocytes. *Chlamydia* is able to infect these innate immune cells, albeit less effectively than epithelial cells [8]. The importance of mononuclear phagocytes (monocytes and macrophages), is reflected in the increased morbidity and mortality of animals selectively depleted of these cells [9,10]. Further, in models of *Chlamydia* infection, a mononuclear infiltrate correlates with a reduced pathological injury [11]. Recent evidence has shown that *Chlamydia* can survive in macrophage albeit much reduced in comparison to epithelial cells [12]. The initial signals to recruit and coordinate these cells remain unclear, but resident immune cells such as macrophages, may play an important role.

Macrophages comprise a significant proportion of cells in healthy tissues [13] and are rapidly differentiated from monocytes recruited during inflammation. It has long been recognized that morphologically and spatially, macrophages represent a heterogeneous population of related cells, however only recently have these differences been functionally classified. Classically activated (M1) macrophages arise from IFNγ/toll-like receptor (TLR) stimulation whereas alternatively activated (M2) macrophages arise from IL-4 stimulation. Consensus has not been reached on the optimum method of detection of macrophage phenotypes, but quantitative real-time PCR (RT-PCR) remains the current gold standard. It is unknown whether these phenotypes represent extremes on a spectrum of activation states, or chronological states of activation [14,15], yet it is an established phenomenon *in vitro* with work ongoing to characterize polarization *in vivo*. Unlike the terminal differentiation seen in adaptive immune cells, polarized macrophages remain relatively plastic in that they are able to take on multiple phenotypes depending on the stimuli, reflecting the broad adaptability of the innate immune system in contrast to the rigid specialization of adaptive immunity.

There is an increasing awareness that macrophage polarization plays an important role in infectious diseases [16,17]. M1 macrophages have been shown to possess bactericidal properties, especially against intracellular pathogens, whereas M2 macrophages support the growth of the same pathogens [18]. Selected intracellular pathogens, such as *Francisella* , have been shown to induce an M2 polarization in macrophages, allowing for their replication at the expense of the host [19]. Although *Chlamydia* has been reported to infect cells of the monocytic lineage, *Chlamydia* ’s effect on macrophage polarization has yet to be explored, and the ability of *Chlamydia* to infect polarized macrophages remains unresolved. Here we show *Chlamydia* does not induce macrophage polarization during intracellular infection as other intracellular bacteria do. We also demonstrate that polarized macrophages have a differential capacity to control *Chlamydia* , with M2 macrophages being permissive to *Chlamydia* growth and M1 macrophages being able to control *Chlamydia* through the induction of persistence.

## Results

### *Chlamydia* infection does not alter the polarization state of non-polarized (M0) macrophages

**Figure 1:**
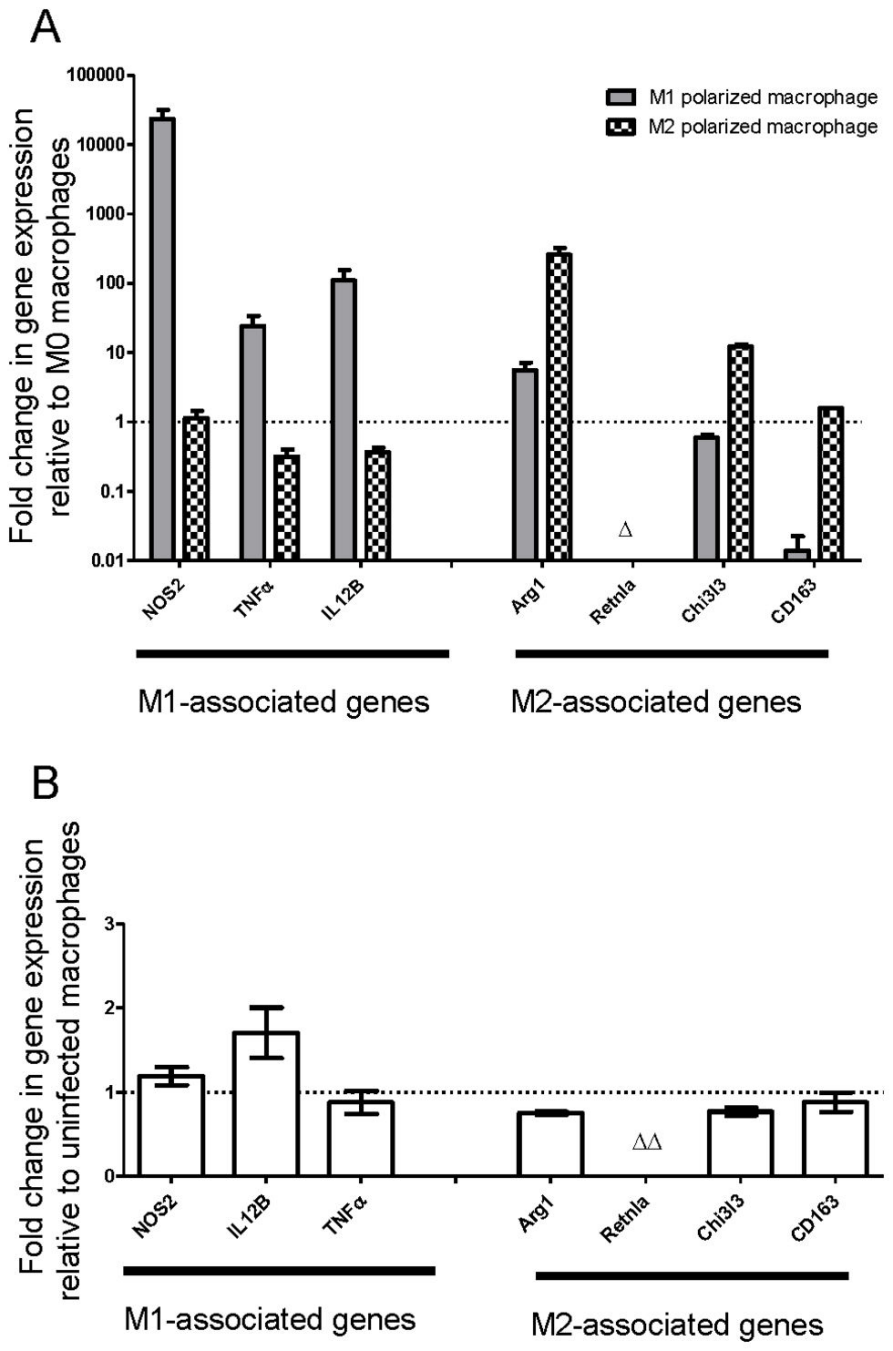
*Chlamydia muridarum* does not induce a gene expression profile of polarization in naïve macrophages. Gene expression profiles determined by RT-PCR of BMDM. (A) Positive controls of polarized BMDM show differential expression in key M1- and M2-associated genes relative to expression seen in M0 macrophages. (∆) denotes detectable *Retnla* expression in M2 macrophages but no detection in M0 or M1 macrophages. (B) M0 macrophages were infected for 24 hr with 1 MOI *Chlamydia* . *16sRNA* detected in infected macrophages by RT-PCR, but not uninfected indicative of bacterial presence. Expression of M1-associated genes and expression of M2-associated genes show no significant alteration with infection as determined by one-sample t-test against hypothetical value of 1.0. (∆∆) denotes no *Retnla* detected in uninfected or uninfected macrophages. Data are averages ±SEM from three independent experiments.

 Based on reports of intracellular pathogens being able to induce an M2 profile in macrophages [17,19], we initially hypothesized that *Chlamydia* would do the same. We assessed the state of macrophage polarization using RT-PCR as this remains the gold standard method. Our positive controls, macrophages polarized to M1 or M2, showed expected expression profiles of key M1 or M2 markers (Figure 1A). Non-polarized bone marrow derived macrophages (BMDM) showed very little difference in the expression of key M1 or M2 genes upon infection compared to the large differences shown in polarized macrophages (Figure 1B). *Chlamydia 16sRNA* was highly expressed in infected macrophages, but was not present in uninfected macrophages.

### Polarized macrophages are not infected equally

**Figure 2:**
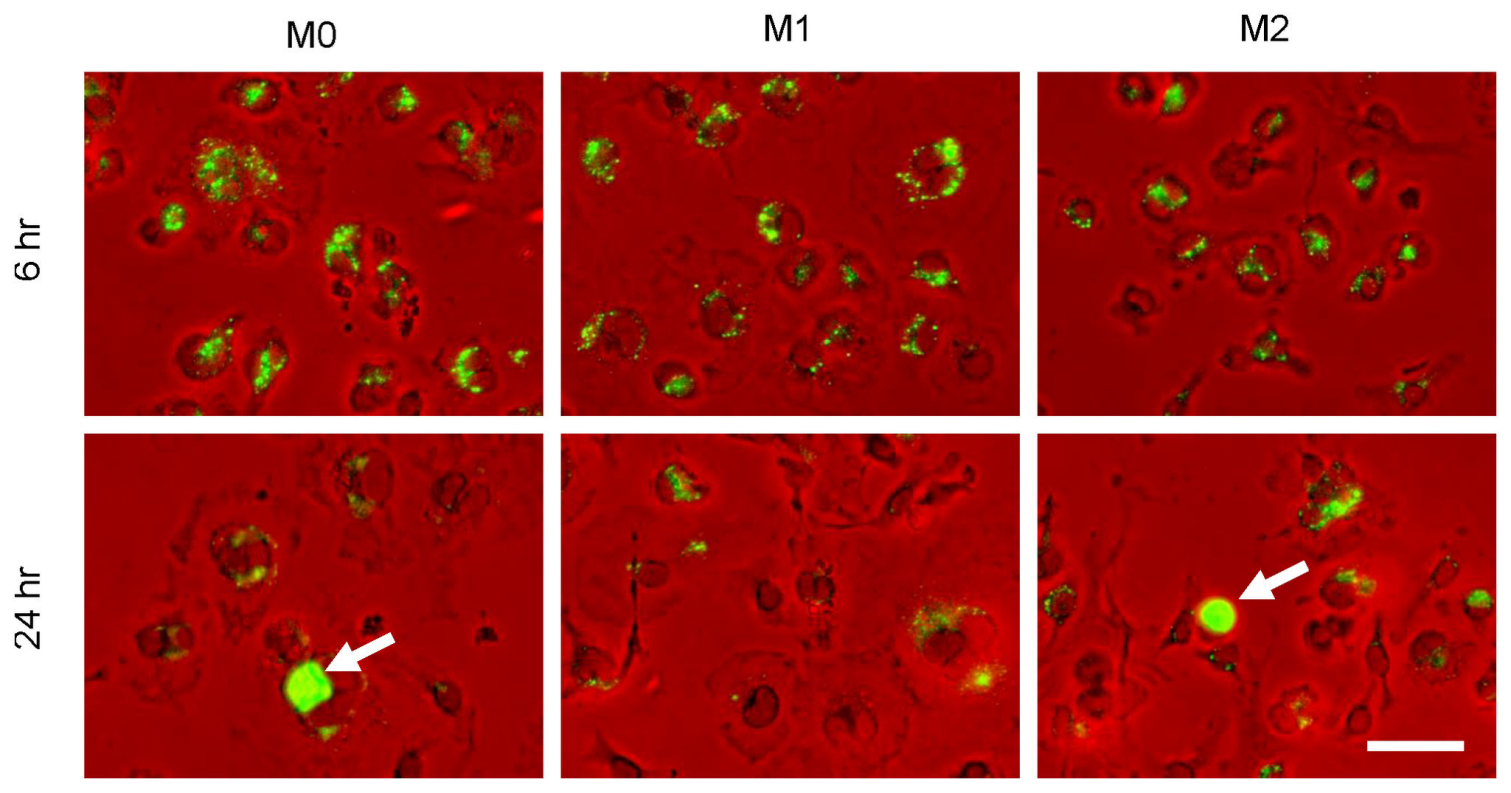
Polarized macrophages are not infected equally. BMDM plated at 2.5 x10^5^ cells/well in 24 well plate and pre-polarized prior to infection for 6–48 hr. Cells fixed and stained with anti-LPS antibody. Representative images of duplicate wells at 6 hr and 24 hr p.i. By 24 hr p.i. large, visible inclusions (white arrows) where seen in M0 and M2 macrophages, but not M1 macrophages. Scale bar is 50µm.

As it is known that M1 macrophages have an enhanced ability to control intracellular bacteria, we examined how pre-polarized primary macrophages may affect the outcome of infection with *Chlamydia* . We infected pre-polarized macrophages with *Chlamydia* over a time course of 6 to 48 hr. At the designated time points we fixed and directly stained the macrophages for *Chlamydia* (Figure 2). At early time points most macrophages contained a number of small inclusions staining for *Chlamydia* . The number of inclusions per cell or number of cells infected appeared independent of macrophage phenotype, although this was not possible to quantify owing to the dispersed nature of the early inclusions. Interestingly, we observed these inclusions to be perinuclear in nature, consistent with previous reports on *Chlamydia* inclusion trafficking to the microtubule organizing centre (MTOC) [20]. At later time points (>24 hr), large inclusions comparable in morphology to those seen during *Chlamydia* growth in fibroblasts, were visible only in M0 and M2 infected macrophages but not M1 macrophages. Small inclusions were still seen in all macrophage phenotypes at these time points, albeit at a lower frequency than at earlier time points. Secondary infections were evident at 48 hr, visible as a cluster of infected cells (data not shown).

**Figure 3:**
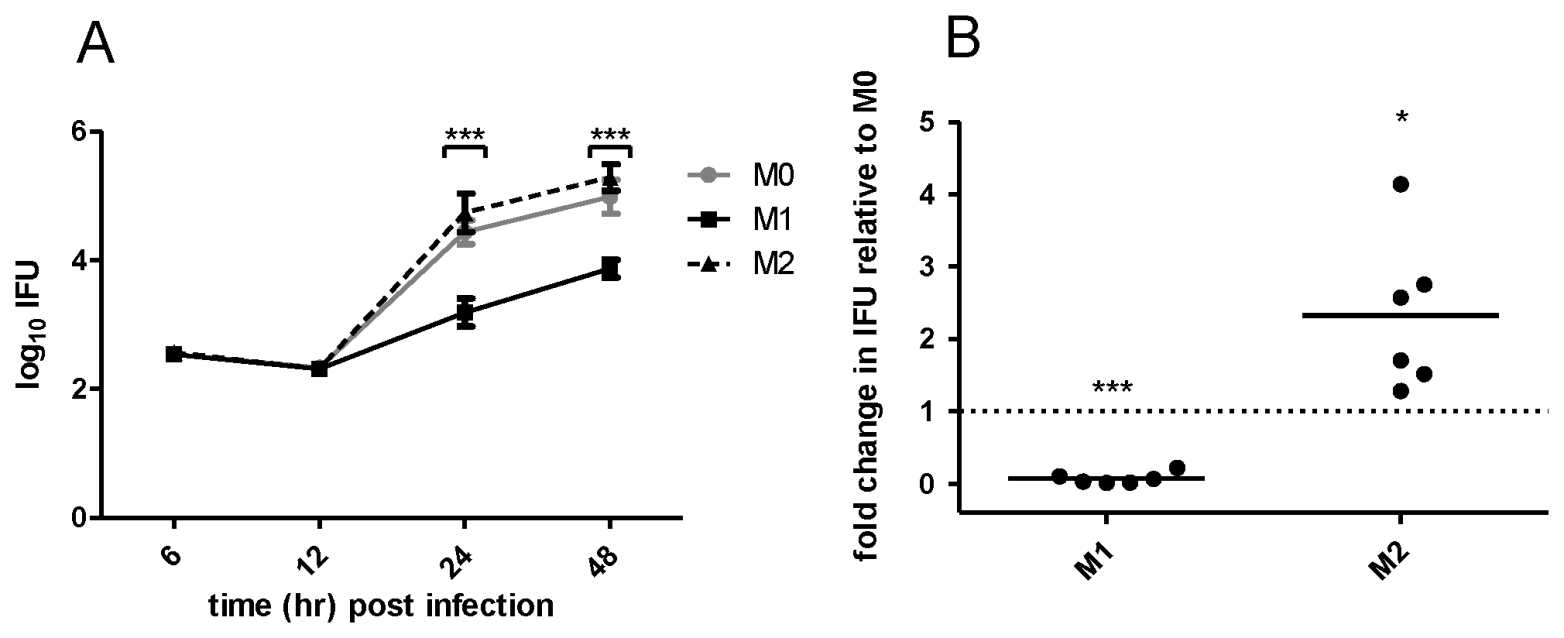
M1 macrophages demonstrate lower *Chlamydia* load than M0 and M2 macrophages. Polarized BMDM were infected for the indicated time points. (A) *Chlamydia* growth seen at 24 and 48 hr p.i. was significantly higher in M0 and M2 macrophages versus M1 macrophages as determined by repeated measures two-way ANOVA with Boneferroni post-test. Data is mean ±SEM from three independent experiments. (B) At 24 hr p.i. M2 macrophages contain significantly more IFU than M0 macrophages whilst M1 macrophages contain less. As inter-experimental IFU differed, IFU in M1 and M2 normalized to that in M0 prior to analysis. Data is mean ±SEM from six independent experiments and analyzed by one-sample t-test against hypothetical value of 1.0.

Enumeration assays were used to quantify the effect of macrophage polarization on *Chlamydia* growth. As mature *Chlamydia* EB escape from infected cells into the supernatant, we harvested the supernatant and cell lysate for enumeration. This however presented a subsequent problem as macrophages release copious cytokines, some of which could affect *Chlamydia* growth during enumeration in fibroblasts. For this reason we performed ultracentrifugation to separate EB from cell debris and supernatants for each BMDM sample prior to enumeration. Using this protocol, we showed a 20-40 fold decrease in inclusion forming units (IFU) after growth in M1 macrophages versus M0 or M2 macrophages respectively (24 hr, p<0.001; 48 hr p<0.001) (Figure 3A). Low levels of *Chlamydia* at 6 and 12 hr post-infection (p.i.) represent residual EB left in each well after washing, since during these time points intracellular *Chlamydia* are expected to be in the non-infectious RB form.

We repeated experiments at 24 hr p.i. with an increased number of individual experiments (Figure 3B) to assess whether the trend of higher IFU in M2 versus M0 macrophages seen in Figure 3A was significant. For each individual experiment we normalized IFU seen in M1 and M2 macrophages to IFU in M0 macrophages to account for inter-experiment variation. This analysis showed M2 macrophages to harbor a significantly higher *Chlamydia* load than M0 macrophages, suggesting enhanced susceptibility to infection.

### RT-PCR demonstrates control of *Chlamydia* by M1 macrophages involves persistence

**Figure 4:**
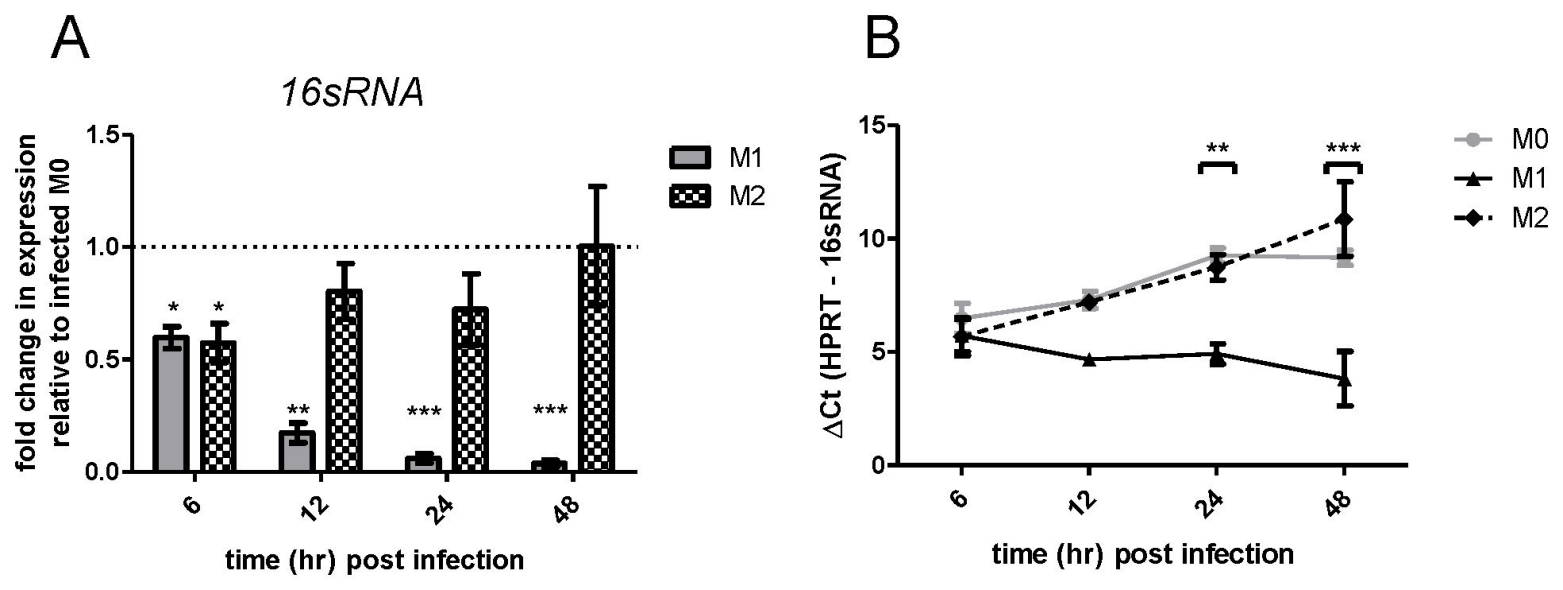
RT-PCR of *Chlamydia 16sRNA* demonstrates that *16sRNA* remains stable in M1 macrophages, but increases in M0 and M2. Polarized BMDM infected with *Chlamydia* were harvested at times indicated. (A) Pfaffl calculated fold change in *16sRNA* in polarized macrophages (M1/M2) versus M0 demonstrates significantly lower *Chlamydia* load in M1 macrophages at all timepoints. Treatments and timepoints analyzed separately by one-sample t-test versus hypothetical mean of 1.0 (B) Difference in ∆Ct between *HPRT* and *16sRNA* for all samples demonstrates that decreased fold change in *16sRNA* in M1 macrophages is due to increased *16sRNA* expression in M0 macrophages rather than a decrease in M1. Data analyzed by repeated measures two-way ANOVA and Bonferroni’s post test. All data is mean ±SEM of three separate experiments.

As measuring *Chlamydia* IFU provides information on the number of viable infectious *Chlamydia* , we examined the state of intracellular *Chlamydia* through RT-PCR. For these experiments any free EB were excluded by removing media and washing cells once with PBS prior to RNA extraction. We initially examined *C. muridarum 16sRNA* expression as an indicator of the number of viable intracellular *Chlamydia* . Relative to that observed in M0 macrophages, *16sRNA* levels were significantly lower in M1 macrophages from 6 to 48 hr p.i. (Figure 4A), whereas *16sRNA* levels remained at similar levels in M2 macrophages compared to M0 macrophages.

As fold change does not distinguish whether the observed change is from increased expression in the control (M0) or reduced expression in the treatment (M1), we re-analyzed this data as delta cycle threshold (∆Ct) (Figure 4B). To minimize error, concentrations of cDNA template were standardized and we kept the Ct constant for both *HPRT* and *16sRNA* for all samples. The results show that *16sRNA*, which was expressed at higher levels than *HPRT* in all samples, was significantly lower in M1 than M0, but no different in M2 than M0. In M1 macrophages ∆Ct *16sRNA* did not change significantly from 6hr timepoint as assessed by repeated measures one-way ANOVA with Dunnett’s post test. This indicates that M0 and M2 macrophages are permissive to intracellular *Chlamydia* growth, whereas M1 macrophages appear to control *Chlamydia* .

**Figure 5:**
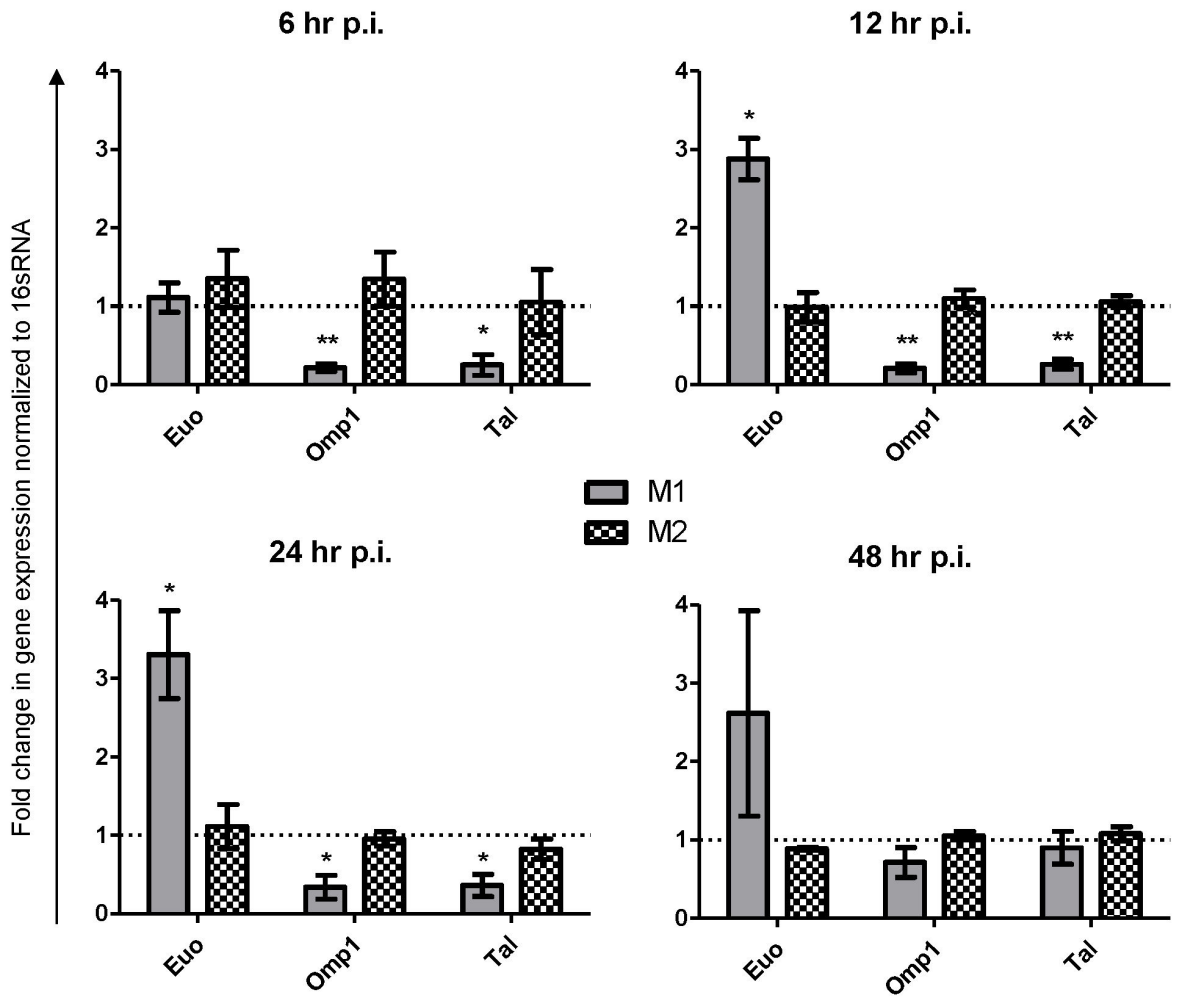
Control of *Chlamydia* by M1 macrophages involves the induction of persistence as demonstrated by RT-PCR. Polarized macrophages were infected for the indicated timepoints. *Chlamydia* gene expression is not altered by growth in M2 macrophages versus growth in M0 macrophages. In M1 macrophages, a gene expression profile characteristic of persistence was seen from 6 hr p.i. infection to 24 hr p.i. whereby *Euo* was up-regulated and *Omp1* and *Tal* were down-regulated. Data is mean ±SEM of three independent experiments and was analyzed using one-sample t-test against hypothetical mean of 1.0.

To further examine the nature of *Chlamydia* control in M1 macrophages we measured bacterial gene expression. To date, persistence in *C. muridarum* has not been assessed through gene expression, so we selected candidate genes based on genes differentially expressed in other species of *Chlamydia* during persistence. Using this approach we screened genes reported to be up-regulated -*Euo, IncA, GroEL2* and *GroEL3* [21,22] - or down-regulated - *omp1*, *omcB*, *FtsK* and *Tal* [23-25]- in M1 and M0 macrophages after 24 hr growth (data not shown). In preliminary experiments *Euo* was consistently up-regulated and *Tal* and *Omp1* were consistently down-regulated so were selected for further experiments. Analysis of the expression of these genes showed significant differential expression during growth in M1 versus M2 macrophages at 6 to 24 hr post infection (Figure 5). At 48 hr p.i. the significance was lost, most likely due to secondary infections seen in M0 macrophages leading to asynchronized infection.

### Electron microscopy (EM) confirms that replication and persistence depend critically on macrophage polarization

**Figure 6:**
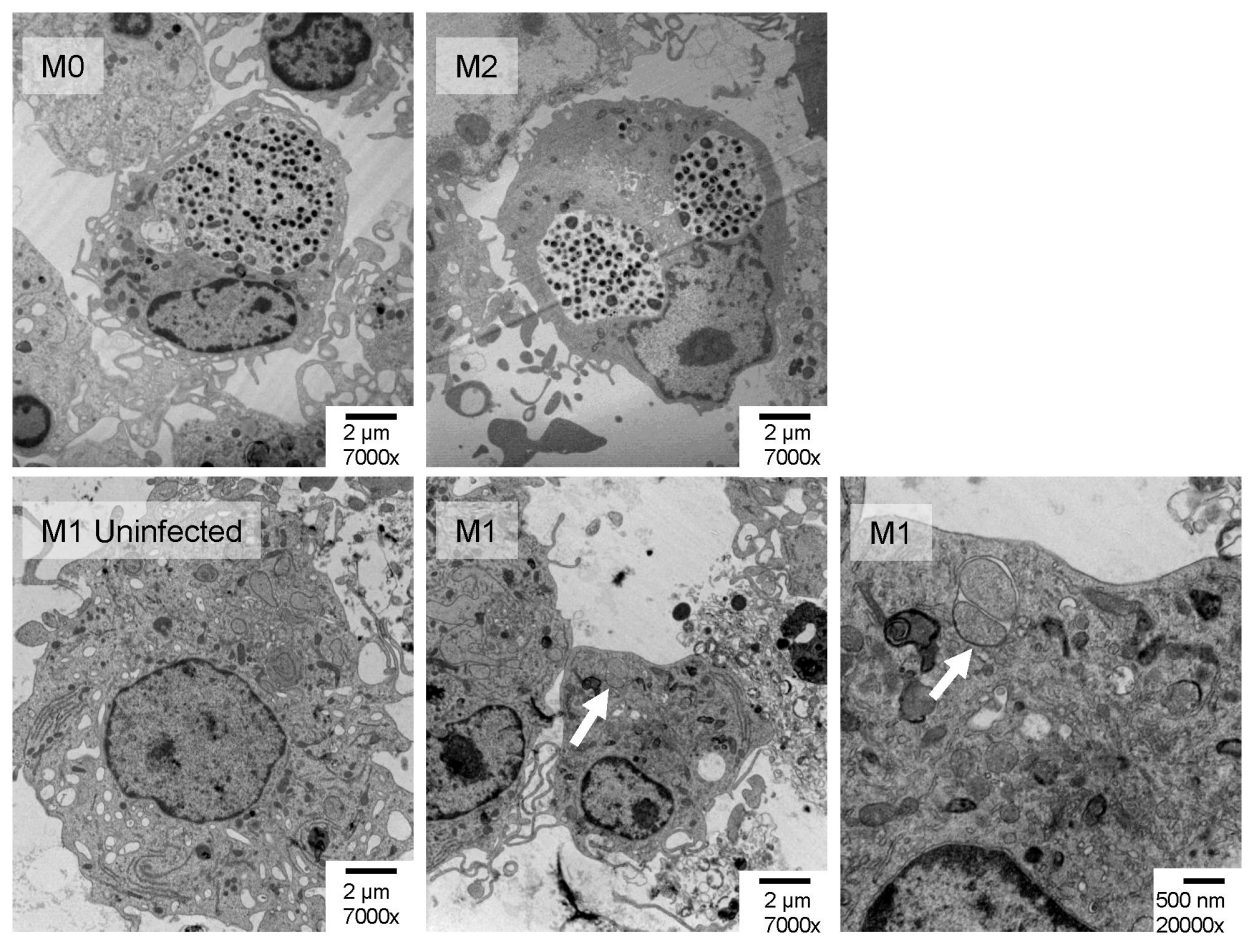
Electron microscopy of infected polarized macrophages demonstrates productive growth in M0 and M2 macrophages but induction of *Chlamydia* persistence in M1 macrophages. Polarized BMDM infected for 24 hr prior to processing for electron microscopy. Mature *Chlamydia* inclusions were seen in M0 and M2 macrophages (top panels). Bottom left panel shows representative uninfected M1 macrophage. Mature inclusions were not seen in infected M1 macrophages. Small inclusions with enlarged amorphous particles, consistent with *Chlamydia* in the persistent state, were seen frequently in infected M1 macrophages (arrows, bottom panels).

Classically, *Chlamydia* persistence was identified through the observation of inclusions with enlarged RB of aberrant morphology. Immunofluorescence staining of *Chlamydia* detects *Chlamydia* antigen, but not live intact *Chlamydia* . For this reason we sought to confirm the profiles of replicative and persistent growth of *Chlamydia* with EM. We examined uninfected macrophages (not shown) and infected macrophages of each phenotype (Figure 6). As previously reported [26], and as observed with direct staining, inclusions within macrophages typically do not fuse to form a large inclusion, rather they tend to remain small with a low number of *Chlamydia* particles. As macrophages are rich with phagosomes and lysosomes, the detection of average smaller inclusions was difficult, however fully mature, enlarged inclusions were seen in M0 and M2 macrophages at a frequency similar to those observed with immunofluorescence and as previously reported [12,27]. Despite extensive scanning of infected M1 macrophage EM sections, such inclusions were not seen, however smaller intracellular vesicles with 1-3 amorphous particles of up to 2 µm in length were observed in most infected, but not uninfected M1 macrophages. We interpret these to be aberrant particles are *Chlamydia* in the persistence state, thus supporting gene expression profiles seen as mentioned above.

## Discussion

An IFNγ-dependant functional innate immune response is absolutely critical in halting exponential growth of *Chlamydia* [7]. As macrophages make up a significant portion of healthy tissue, it is of importance to study the acute host–pathogen interactions between *Chlamydia* and these cells, as such an interaction could have a profound impact in the failure or success of a subsequent immune response to *Chlamydia* . Such a host–pathogen interaction has not yet been studied in the context of macrophage polarization.

Recently, it has been shown that selected intracellular pathogens are able to induce an M2 phenotype in macrophages [19], a mechanism hypothesized to contribute to survival of such microbes. As *Chlamydia* has been shown to survive in mononuclear phagocytes [8], we initially hypothesized such an M2 induction would be operational. On the contrary, *Chlamydia* caused no up-regulation in M2 genes, and surprisingly, no significant alteration in the expression of M1 genes. It appears that *Chlamydia* can infect non-stimulated macrophages without inducing a global anti-bacterial response. This is a novel observation. It is possible that pathogen-associated molecular patterns derived from *Chlamydia* remain hidden within the inclusion and that macrophages only become activated to an M1 state upon recognition of extracellular *Chlamydia* . It is also possible that macrophage activation is induced by the infection of permissive bystander cells. Further experiments need to be performed to test these hypotheses.

*In situ*, tissue macrophages are unlikely to be in the naïve, unstimulated state, rather they are likely to fall somewhere in the continuum of polarization [15]. Thus examining the ability of *Chlamydia* to infect and survive in macrophages pre-polarized to the extremes of the polarization spectrum presents as a more physiological approach to study *Chlamydia* – macrophage interactions *in vitro*. Although not in the context of macrophage polarization, previous studies have examined the ability of monocytic cell lines to control *Chlamydia* when activated with IFNγ and/or LPS. IFNγ/LPS stimulated murine RAW264.7 cells were shown to limit *C. trachomatis* serovar D in a nitrite-dependant manner [28] and IFNγ stimulation of human monomac 6 cells have been demonstrated to control the growth of *C. pneumoniae* [29]. These results must be interpreted with caution since cell lines are often quite different from their primary cell counterparts. Further, although the murine and human IFNγ response has the same global effect of halting *Chlamydia* infection, it is achieved through different molecular mechanisms; *Chlamydia* has evolved to evade its specific host’s IFNγ response and indeed cross infection of *Chlamydia* to another host species results in an ineffective infection [30].

Here we use a murine pathogen in murine primary cells to overcome the aforementioned obstacles. As demonstrated by immunostaining, we saw equal uptake of *Chlamydia* between all classes of macrophages. Although the number of cells with inclusions diminished in all polarized macrophage states, enlarged mature inclusions were never seen in the M1 macrophage, but were seen in a limited number of M0 and M2 macrophages. This indicates that all macrophages can become infected and all have an inherent ability to control *Chlamydia* , yet through a stochastic process some macrophages remain infected. Further to this, the increase in IFU over time in M1 macrophages, indicates *Chlamydia* can grow productively in these cells, yet this growth is atypical in that large inclusions were not observed. The trend of increasing IFU from 24 to 48 hr p.i. in all macrophage phenotypes suggests a slow but continuous growth of *Chlamydia* inside macrophages as oppose to the rapid and abrupt growth in epithelial and fibroblast cells.

Intriguingly, we also demonstrated that the M2 macrophage is more susceptible to *Chlamydia* infection than their non-polarized counterparts. This has not been reported previously, and could be explained by increased expression of the mannose receptor on M2 macrophages [31], which has been shown to facilitate the uptake of certain species and serovars of *Chlamydia* [32]. This observation could also be due to the reported plasticity of macrophage polarization [33]; it may take longer for M2 polarized macrophages to activate antimicrobial mechanisms than unstimulated M0 macrophages.

We demonstrate that the M1 macrophage is able to cap *Chlamydia* growth. Our *16sRNA* results served to reinforce observations of *Chlamydia* growth seen with direct staining and with IFU assessment. At 6 hr post-infection *16sRNA* were similar in all macrophage phenotypes with significance depending on method of analysis, supporting direct staining observations of equal uptake. As early as 12 hr, there were trends of growth in M0 and M2 macrophages as demonstrated by increased ∆Ct *16sRNA*, while *16sRNA* in M1 macrophages did not change.

With IFU and *16sRNA* suggesting persistence in the M1 macrophage we sought to confirm this with *Chlamydia* gene expression. Here we report the up-regulation of *Euo*, a putative repressor of EB genes [34], to be up-regulated during persistence in *C. muridarum*. We also report *Tal* and *Omp1*, which code for metabolism and outer membrane proteins respectively, to be down-regulated. These three genes are similarly differentially regulated during persistence of other species of *Chlamydia* [21,35]. Our genetic characterization focused on three genes in particular, but it is not surprising that the other genes examined, as reported in the methods section, did not show significant alterations in expression. Candidate genes, some of which were putative genes in the published *C. muridarum* genome, were based on genes differentially expressed upon other inducers of persistence, in other species of *Chlamydia* . It is established that the gene expression profile of persistence changes depending on these variables [5].

Our EM studies confirm viable growth in M0 and M2 macrophages, and lend support to the concept of persistence in M1 macrophages. This EM analysis is confounded by inclusions within monocytic cells typically being small and disperse as reported [26], and by the highly convoluted plasma membranes and large numbers of inclusion-like phagosomes and electron dense, EB-like lysosomes in macrophages. It is difficult to state unequivocally that the large amorphous particles seen within inclusions in M1 macrophages are persistent *Chlamydia* , however the morphology is definitely close that to *C. trachomatis* and *C. pneumoniae* persistent particles [36].

To the best of our knowledge this is the first conclusive evidence, and the first genetic profiling, of the phenomenon of persistence in *C. muridarum*. Previously, Rey-Ladino et al. [37] had reported *C. muridarum* persistence in murine dendritic cells, however these observations were circumstantial with *Chlamydia* being observed through direct staining coupled with minimal re-culture. Our findings lay the groundwork for a more comprehensive analysis of host–pathogen interactions employing *C. muridarum* infection in mice as a model for human disease.

In conclusion, this study sheds light on the central role of macrophage polarization in the control of *Chlamydia* infection. The role IFNγ plays in the control of *Chlamydia* is supported by our study, yet we demonstrate how IFNγ is a double-edged sword by contributing to the induction of persistence. The ability of *Chlamydia* to survive in what would appear the most inhospitable of the innate immune cells is likely sculpted by thousands of years of host–pathogen interaction crafting an effective parasite. Perhaps the optimum response to *Chlamydia* infection the host can achieve is control by the induction of persistence. Indeed, *Chlamydia* may lay latent in an individual for decades before re-activation [38]. Further work needs to be done to examine whether the effects seen in this study hold true for human *Chlamydia* infecting polarized human macrophages. Finally, the differential infection of M1 and M2 macrophages may have profound impacts on the outcome to *Chlamydia* infections due to the genetic background of the host: C57Bl/6 mice, which are less susceptible to *Chlamydia* , are known to have a Th1 immune predominance in comparison with BALBc mice [39]. This could shed light on why patients with *Chlamydia* sequelae have a predominantly Th2 response to the organism [40,41].

## Materials and Methods

### Ethics statement

All experiments were approved by the Animal Resource Centre of the University Health Network (UHN), Toronto (Animal Use Protocol #1072). UHN maintains an animal care and use program certified by the Canadian Council on Animal Care (CCAC) and all procedures are conducted in accordance with guidelines in the Province of Ontario’s Animals for Research Act.

### *Chlamydia muridarum*

All cells were incubated at 37° C, 5% CO_2_. McCoy B fibroblasts (ATCC) were passaged in αMEM (Invitrogen) supplemented with 10% FCS (Invitrogen), 10 µg/ml gentamicin (Sigma) and 25 µg/ml vancomycin (Sigma). *C. muridarum* (ATCC) was cultured in McCoy B cells in this media further supplemented with 1 µg/ml cycloheximide (Sigma) and 8.8% d-glucose (Sigma) as previously reported [42]. *Chlamydia* EB were isolated by density ultracentrifugation using a 50%-20% Gastrografin gradient (Roche) and were stored in sucrose phosphate-glutamic acid buffer (8.5 mM Na_2_HPO_4_, 4 mM NaH_2_PO_4_, 220 mM sucrose, 0.5 mM l-glutamic acid, pH 7.4 [Sigma]) at -80° C prior to use. IFU of purified EB was determined through an enumeration assay as described below.

### Animals and Bone Marrow Isolation

L-cells (a gift from Dr. F. Tsui, originally from ATCC) were plated at 5 x10^5^ cells/75 cm^2^ flask (Corning) in 25 ml RPMI 1640 (Invitrogen) with 10 µg/ml gentamicin, 25 µg/ml vancomycin, 50 µM mercaptoethanol and cultured for one week after which L-cell-conditioned media was harvested, filtered and frozen at -20° C. Bone marrow was harvested from femurs and tibias of 8-12 week old male BALB/c mice. Mice were obtained from Jackson Laboratories and housed in a 12 hr light/dark cycle under SPF conditions as mandated by the Canadian Council on Animal Care.

Bone marrow was differentiated into macrophages using supplemented RPMI and 20% L-cell conditioned media as previously described [43]. Macrophage purity was routinely >95% as assessed by flow cytometry for CD11b and F480 (data not shown).

### Bone marrow derived macrophage (BMDM) polarization, infection and repolarization

BMDM were plated in 24-well plates (Corning) in bone marrow media without L-cell conditioned media, at a density of 5 x10^5^ cells/well unless indicated. These cells were incubated for 10-12 hr prior to infection without treatment for non-polarized (M0) or BMDM were polarized to either M1 macrophages with 20 ng/ml IFNγ (RnD Systems) and 100 ng/ml LPS (Sigma) or to M2 macrophages with 20 ng/ml IL-4 (RnD Systems) as reported [44]. Polarization of macrophages in our hands matched that reported in the literature [15,45] as assessed by supernatant nitrite, arginase activity, cytokine release (Figure S1 in Supporting Information S1) and gene expression (Figure 1A). We unexpectedly found IL-10 to be upregulated in M1 macrophages but not M2 macrophages, however this has been reported in the literature [46]. For infection with *Chlamydia* , media was removed and 1 multiplicity of infection (MOI) was added. The plates were spun at 600 g for 20 min at 37° C to synchronize infection. The supernatant was replaced to remove free *Chlamydia* . As macrophage polarization is believed to be a plastic process, cytokines were replaced after infection and left in media for the duration of experiments. LPS was not replaced as prolonged exposure (>16 hr) can induce M2 polarization of macrophages due to endotoxin tolerance [47].

### Enumeration assay

For enumeration of intracellular and extracellular viable infectious *Chlamydia* , infected wells were first scraped to lyse BMDM. Cell lysate in media was harvested and frozen at -80° C. McCoy B cells were plated at 2.5 x10^5^ cells/well in 24-well plates overnight to generate confluent monolayers. Lysate was thawed at 37° C and sonicated for 15 sec. The lysate was spun at 600 g for 5 min to remove cellular debris, prior to sterile ultracentrifugation at 19,500 g (L80-70m, Beckman Coulter) for 30 min at 4C to separate *Chlamydia* from conditioned media. The *Chlamydia* pellet was serially diluted and McCoy B cells were infected by centrifugation as above. Media was not removed and McCoy B cells were incubated for 24 hr prior to counting of infected cells.

### Immunofluorescent microscopy

For direct staining of *Chlamydia* in BMDM or McCoy B cells, FITC-conjugated, anti-LPS antibody was used as per the manufacturer’s recommendations (Biorad). Stained samples were viewed with Nikon ECLipse TE2000U. ImageJ (NIH) was used to merge phase contrast and fluorescent images. For enumeration assays, the number of inclusions per well were counted whereby one infected cell was considered to be one inclusion forming unit (IFU).

### RT-PCR

RNA was extracted from cells using TRIzol (Invitrogen) and was reverse transcribed using random primers and superscript II (Invitrogen). RT-PCR was performed with an ABI 7900HT (Applied Biosystems) system using power SYBR green (Applied Biosystems). RT-PCR data was analyzed with SDS 2.4.2 (Applied Biosystems). Primers for RT-PCR were designed with Primer Express (Applied Biosystems), tested for intra- and inter-species cross-reactivity using primerBLAST (NCBI) and ordered from ACGT corp (Figure S2 in Supporting Information S1). Primer efficiencies were assessed through construction of serial dilution standard curves and were between 90 and 105%. Multiple housekeeper genes were initially assessed, and HPRT was selected as it proved to be unaffected by polarization or *Chlamydia* infection (data not shown). *16sRNA* is a commonly used housekeeper for *Chlamydia* [21] and does not appear to be differentially expressed during stress [48]. Pfaffl equation was used to calculate fold-change gene of interest in comparing treatments to controls [49].

### Electron microscopy

For electron microscopy, BMDM were plated at 2 x10^6^ cells/well in 6 well plates (Corning) and were subsequently polarized and infected as above. For harvesting, macrophages were washed once with PBS (Invitrogen) and treated for 10 min with accutase (Sigma). Cells were flushed prior to fixation to dislodge from plate and processed as previously described [50]. A Hitachi H7000 electron microscope with XR-60 camera (AMT Co.) was used to examine specimens.

### Statistics

GraphPad Prism 5 (TreeStar) was used for the generation of all graphs and statistical analysis. All data was expressed as mean with standard error of mean (SEM) of at least three independent experiments. To calculate statistical significance of fold changes, a one-sample t-test was used versus a hypothetical mean of 1.0, with this value representing no change in treatment relative to control. For time course analyses, repeated measures two-way ANOVA was used with Bonferroni’s post test. For all graphs: * p = 0.01–0.05, ** p = 0.001–0.01, *** p < 0.001.

## Supporting Information

Supporting Information S1 (DOC)

## References

[1] Horn M, Collingro A, Schmitz-Esser S, Beier CL, Purkhold U et al. (2004) Illuminating the evolutionary history of chlamydiae. Science 304: 728-730. doi:10.1126/science.1096330. PubMed: 15073324. 15073324 10.1126/science.1096330

[2] World Health Organization, Geneva Switzerland. (p. 2003) Alliance for the global elimination of blinding trachoma by 2020

[3] Read TD, Brunham RC, Shen C, Gill SR, Heidelberg JF et al. (2000) Genome sequences of chlamydia trachomatis MoPn and chlamydia pneumoniae AR39. Nucleic Acids Res 28: 1397-1406. doi:10.1093/nar/28.6.1397. PubMed: 10684935. 10684935 10.1093/nar/28.6.1397 PMC111046

[4] AbdelRahman YM, Belland RJ (2005) The chlamydial developmental cycle. FEMS Microbiol Rev 29: 949-959. doi:10.1016/j.femsre.2005.03.002. PubMed: 16043254. 16043254 10.1016/j.femsre.2005.03.002

[5] Hogan RJ, Mathews SA, Mukhopadhyay S, Summersgill JT, Timms P (2004) Chlamydial persistence: Beyond the biphasic paradigm. Infect Immun 72: 1843-1855. doi:10.1128/IAI.72.4.1843-1855.2004. PubMed: 15039303. 15039303 10.1128/IAI.72.4.1843-1855.2004 PMC375192

[6] Rottenberg ME, Gigliotti-Rothfuchs A, Wigzell H (2002) The role of IFN-γ in the outcome of chlamydial infection. Curr Opin Immunol 14: 444-451. doi:10.1016/S0952-7915(02)00361-8. PubMed: 12088678. 12088678 10.1016/s0952-7915(02)00361-8

[7] Rottenberg ME, Rothfuchs AG, Gigliotti D, Ceausu M, Une C et al. (2000) Regulation and role of IFN-γ in the innate resistance to infection with chlamydia pneumoniae. J Immunol 164: 4812-4818. PubMed: 10779789. 10779789 10.4049/jimmunol.164.9.4812

[8] Beagley KW, Huston WM, Hansbro PM, Timms P (2009) Chlamydial infection of immune cells: Altered function and implications for disease. Crit Rev Immunol 29: 275-305. doi:10.1615/CritRevImmunol.v29.i4.10. PubMed: 19673684. 19673684 10.1615/critrevimmunol.v29.i4.10

[9] Miyairi I, Ramsey KH, Patton DL (2010) Duration of untreated chlamydial genital infection and factors associated with clearance: Review of animal studies. J Infect Dis 201: S96-S103. doi:10.1086/652393. PubMed: 20470047. 20470047 10.1086/652393

[10] Qiu H, Fan Y, Joyee AG, Wang S, Han X et al. (2008) Type I IFNs enhance susceptibility to chlamydia muridarum lung infection by enhancing apoptosis of local macrophages. J Immunol 181: 2092-2102. PubMed: 18641348. 18641348 10.4049/jimmunol.181.3.2092

[11] Inman RD, Chiu B (2006) Early cytokine profiles in the joint define pathogen clearance and severity of arthritis in chlamydia-induced arthritis in rats. Arthritis Rheum 54: 499-507. doi:10.1002/art.21643. PubMed: 16447224. 16447224 10.1002/art.21643

[12] Sun HS, Eng EWY, Jeganathan S, Sin AT-, Patel PC, et al. (2012) Chlamydia trachomatis vacuole maturation in infected macrophages. J Leukoc Biol 92: 815-827

[13] Gordon S, Taylor PR (2005) Monocyte and macrophage heterogeneity. Nat Rev Immunol 5: 953-964. doi:10.1038/nri1733. PubMed: 16322748. 16322748 10.1038/nri1733

[14] Gordon S, Martinez FO (2010) Alternative activation of macrophages: Mechanism and functions. Immunity 32: 593-604. doi:10.1016/j.immuni.2010.05.007. PubMed: 20510870. 20510870 10.1016/j.immuni.2010.05.007

[15] Mosser DM, Edwards JP (2008) Exploring the full spectrum of macrophage activation. Nat Rev Immunol 8: 958-969. doi:10.1038/nri2448. PubMed: 19029990. 19029990 10.1038/nri2448 PMC2724991

[16] Murray PJ, Wynn TA (2011) Protective and pathogenic functions of macrophage subsets. Nat Rev Immunol 11: 723-737. doi:10.1038/nri3073. PubMed: 21997792. 21997792 10.1038/nri3073 PMC3422549

[17] Benoit M, Desnues B, Mege JL (2008) Macrophage polarization in bacterial infections. J Immunol 181: 3733-3739. PubMed: 18768823. 18768823 10.4049/jimmunol.181.6.3733

[18] Kahnert A, Seiler P, Stein M, Bandermann S, Hahnke K et al. (2006) Alternative activation deprives macrophages of a coordinated defense program to mycobacterium tuberculosis. Eur J Immunol 36: 631-647. doi:10.1002/eji.200535496. PubMed: 16479545. 16479545 10.1002/eji.200535496

[19] Shirey KA, Cole LE, Keegan AD, Vogel SN (2008) Francisella tularensis live vaccine strain induces macrophage alternative activation as a survival mechanism. J Immunol 181: 4159-4167. PubMed: 18768873. 18768873 10.4049/jimmunol.181.6.4159 PMC2637804

[20] Scidmore MA (2011) Recent advances in chlamydia subversion of host cytoskeletal and membrane trafficking pathways. Microbes Infect 13: 527-535. doi:10.1016/j.micinf.2011.02.001. PubMed: 21334451. 21334451 10.1016/j.micinf.2011.02.001 PMC3092832

[21] Belland RJ, Zhong G, Crane DD, Hogan D, Sturdevant D et al. (2003) Genomic transcriptional profiling of the developmental cycle of chlamydia trachomatis. Proc Natl Acad Sci U S A 100: 8478-8483. doi:10.1073/pnas.1331135100. PubMed: 12815105. 12815105 10.1073/pnas.1331135100 PMC166254

[22] Gérard HC, Whittum-Hudson JA, Schumacher HR Jr., Hudson AP (2004) Differential expression of three chlamydia trachomatis hsp60-encoding genes in active vs. persistent infections. Microb Pathog 36: 35-39. doi:10.1016/j.micpath.2003.08.005. PubMed: 14643638. 14643638 10.1016/j.micpath.2003.08.005

[23] Gérard HC, Köhler L, Branigan PJ, Zeidler H, Schumacher HR et al. (1998) Viability and gene expression in chlamydia trachomatis during persistent infection of cultured human monocytes. Med Microbiol Immunol (Berl) 187: 115-120. doi:10.1007/s004300050082. PubMed: 9832326. 9832326 10.1007/s004300050082

[24] Gérard HC, Krauße-Opatz B, Wang Z, Rudy D, Rao JP et al. (2001) Expression of chlamydia trachomatis genes encoding products required for DNA synthesis and cell division during active versus persistent infection. Mol Microbiol 41: 731-741. doi:10.1046/j.1365-2958.2001.02550.x. PubMed: 11532140. 11532140 10.1046/j.1365-2958.2001.02550.x

[25] Kokab A, Jennings R, Eley A, Pacey AA, Cross NA (2010) Analysis of modulated gene expression in a model of interferon-γ-induced persistence of chlamydia trachomatis in HEp-2 cells. Microb Pathog 49: 217-225. doi:10.1016/j.micpath.2010.06.002. PubMed: 20558272. 20558272 10.1016/j.micpath.2010.06.002

[26] Airenne S, Surcel H-, Alakärppä H, Laitinen K, Paavonen J, et al (1999) Chlamydia pneumoniae infection in human monocytes. Infect Immun 67: 1445-1449. PubMed: 10024593. 10024593 10.1128/iai.67.3.1445-1449.1999 PMC96479

[27] Nettelnbreker E, Zeidler H, Bartels H, Dreses-Werringloer U, Däubener W et al. (1998) Studies of persistent infection by chlamydia trachomatis serovar K in TPA-differentiated U937 cells and the role of IFN-γ. J Med Microbiol 47: 141-149. doi:10.1099/00222615-47-2-141. PubMed: 9879957. 9879957 10.1099/00222615-47-2-141

[28] Chen B, Stout R, Campbell WF (1996) Nitric oxide production: A mechanism of chlamydia trachomatis inhibition in interferon-γ-treated RAW264.7 cells. FEMS Immunol Med Microbiol 14: 109-120. doi:10.1111/j.1574-695X.1996.tb00277.x. PubMed: 8809546. 8809546 10.1111/j.1574-695X.1996.tb00277.x

[29] Mannonen L, Kamping E, Penttilä T, Puolakkainen M (2004) IFN-γ induced persistent chlamydia pneumoniae infection in HL and mono mac 6 cells: Characterization by real-time quantitative PCR and culture. Microb Pathog 36: 41-50. doi:10.1016/j.micpath.2003.09.001. PubMed: 14643639. 14643639 10.1016/j.micpath.2003.09.001

[30] Coers J, Starnbach MN, Howard JC (2009) Modeling infectious disease in mice: Co-adaptation and the role of host-specific IFNγ responses. PLOS Pathog 5: e1000333. 19478881 10.1371/journal.ppat.1000333 PMC2682201

[31] Stein M, Keshav S, Harris N, Gordon S (1992) Interleukin 4 potently enhances murine macrophage mannose receptor activity: A marker of alternative immunologic macrophage activation. J Exp Med 176: 287-292. doi:10.1084/jem.176.1.287. PubMed: 1613462. 1613462 10.1084/jem.176.1.287 PMC2119288

[32] Kuo C-, Puolakkainen M, Lin T-, Witte M, Campbell LA (2002) Mannose-receptor positive and negative mouse macrophages differ in their susceptibility to infection by chlamydia species. Microb Pathog 32: 43-48. doi:10.1006/mpat.2001.0479. PubMed: 11782120. 11782120 10.1006/mpat.2001.0479

[33] Murray PJ, Wynn TA (2011) Obstacles and opportunities for understanding macrophage polarization. J Leukoc Biol 89: 557-563. doi:10.1189/jlb.0710409. PubMed: 21248152. 21248152 10.1189/jlb.0710409 PMC3058818

[34] Zhang L, Douglas AL, Hatch TP (1998) Characterization of a chlamydia psittaci DNA binding protein (EUO) synthesized during the early and middle phases of the developmental cycle. Infect Immun 66: 1167-1173. PubMed: 9488410. 9488410 10.1128/iai.66.3.1167-1173.1998 PMC108030

[35] Gérard HC, Freise J, Wang Z, Roberts G, Rudy D et al. (2002) Chlamydia trachomatis genes whose products are related to energy metabolism are expressed differentially in active vs. persistent infection. Microbes Infect 4: 13-22. doi:10.1016/S1286-4579(01)01504-0. PubMed: 11825770. 11825770 10.1016/s1286-4579(01)01504-0

[36] Wyrick PB (2010) Chlamydia trachomatis persistence in vitro: An overview. J Infect Dis 201: S88-S95. doi:10.1086/652394. PubMed: 20470046. 20470046 10.1086/652394 PMC2878585

[37] Rey-Ladino J, Jiang X, Gabel BR, Shen C, Brunham RC (2007) Survival of chlamydia muridarum within dendritic cells. Infect Immun 75: 3707-3714. doi:10.1128/IAI.01618-06. PubMed: 17502393. 17502393 10.1128/IAI.01618-06 PMC1952003

[38] Dean D, Suchland RJ, Stamm WE (2000) Evidence for long-term cervical persistence of chlamydia trachomatis by omp1 genotyping. J Infect Dis 182: 909-916. doi:10.1086/315778. PubMed: 10950788. 10950788 10.1086/315778

[39] Jiang X, Shen C, Yu H, Karunakaran KP, Brunham RC (2010) Differences in innate immune responses correlate with differences in murine susceptibility to chlamydia muridarum pulmonary infection. Imm 129: 556-566. doi:10.1111/j.1365-2567.2009.03157.x. PubMed: 20102413. 10.1111/j.1365-2567.2009.03157.x PMC2842502 20102413

[40] Holland MJ, Bailey RL, Conway DJ, Culley F, Miranpuri G et al. (1996) T helper type-1 (Th1)/Th2 profiles of peripheral blood mononuclear cells (PBMC); responses to antigens of chlamydia trachomatis in subjects with severe trachomatous scarring. Clin Exp Immunol 105: 429-435. doi:10.1046/j.1365-2249.1996.d01-792.x. PubMed: 8809130. 8809130 10.1046/j.1365-2249.1996.d01-792.x PMC2200527

[41] Yin Z, Braun J, Neure L, Peihua WU, Liu L et al. (1997) Crucial role of interleukin-10/interleukin-12 balance in the regulation of the type 2 T helper cytokine response in reactive arthritis. Arthritis Rheum 40: 1788-1797. doi:10.1002/art.1780401010. PubMed: 9336412. 9336412 10.1002/art.1780401010

[42] Mukhopadhyay S, Clark AP, Sullivan ED, Miller RD, Summersgill JT (2004) Detailed protocol for purification of chlamydia pneumoniae elementary bodies. J Clin Microbiol 42: 3288-3290. doi:10.1128/JCM.42.7.3288-3290.2004. PubMed: 15243095. 15243095 10.1128/JCM.42.7.3288-3290.2004 PMC446299

[43] Weischenfeldt J, Porse B (2008) Bone marrow-derived macrophages (BMM): Isolation and applications. Cold Spring Harb Protoc 2008: pdb.prot5080. 10.1101/pdb.prot5080 21356739

[44] Martinez FO, Gordon S, Locati M, Mantovani A (2006) Transcriptional profiling of the human monocyte-to-macrophage differentiation and polarization: New molecules and patterns of gene expression. J Immunol 177: 7303-7311. PubMed: 17082649. 17082649 10.4049/jimmunol.177.10.7303

[45] Mantovani A, Sica A, Sozzani S, Allavena P, Vecchi A et al. (2004) The chemokine system in diverse forms of macrophage activation and polarization. Trends Immunol 25: 677-686. doi:10.1016/j.it.2004.09.015. PubMed: 15530839. 15530839 10.1016/j.it.2004.09.015

[46] Lopez-Castejón G, Baroja-Mazo A, Pelegrín P (2010) Novel macrophage polarization model: From gene expression to identification of new anti-inflammatory molecules. Cell Mol Life Sci 68: 3095-3107. PubMed: 21188461. 21188461 10.1007/s00018-010-0609-y PMC11114961

[47] Lawrence T, Natoli G (2011) Transcriptional regulation of macrophage polarization: Enabling diversity with identity. Nat Rev Immunol 11: 750-761. doi:10.1038/nri3088. PubMed: 22025054. 22025054 10.1038/nri3088

[48] Mäurer AP, Mehlitz A, Mollenkopf HJ, Meyer TF (2007) Gene expression profiles of chlamydophila pneumoniae during the developmental cycle and iron depletion-mediated persistence. PLOS Pathog 3: 0752-0769. PubMed: 17590080. 10.1371/journal.ppat.0030083 PMC1894823 17590080

[49] Pfaffl MW (2001) A new mathematical model for relative quantification in real-time RT-PCR. Nucleic Acids Res 29: e45. doi:10.1093/nar/29.9.e45. PubMed: 11328886. 11328886 10.1093/nar/29.9.e45 PMC55695

[50] Lewis PR, Knight DP (1977) Cytological staining methods in electron microscopy. In: Glauert AM Staining: Methods for Sectioned Material Amsterdam, Netherlands; Elsevier, North-Holland Biomedical Press. pp. 49 -52

