# Supplementary material for: Intracellular Survival and Persistence of Chlamydia muridarum Is Determined by Macrophage Polarization
Source: PLoS One. 2013 Aug 14;8(8):e69421. doi: 10.1371/journal.pone.0069421 (PMC3743904; doi:10.1371/journal.pone.0069421)
Supplement: Supporting Information S1 — (DOC) [file pone.0069421.s001.doc]

# Supplementary Figures

**
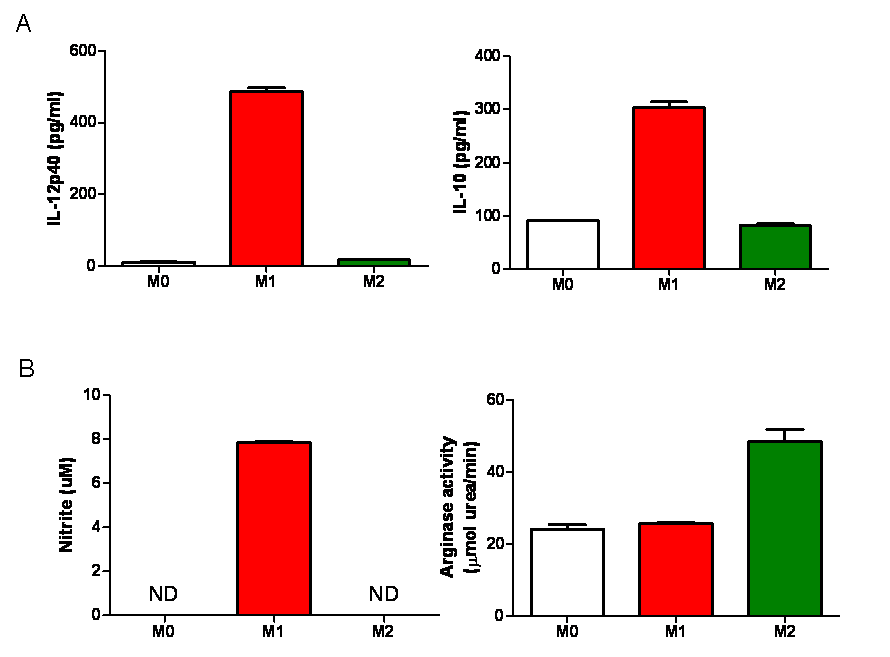
**

**Figure S1: Bone marrow derived macrophages (BMDM) are polarized after 12 hr exposure to M1 or M2 conditions.** Bone marrow was harvest from 8-12 week old male Balb/c mice and differentiated to macrophages (CD11b+F4/80+) using L-cell conditioned media. BMDM were then left unpolarized (M0), or polarized to M1 (IFNy/LPS) or M2 (IL-4) for 12 hr as described in the methods section. (A) Supernatants at 12 hr polarization tested for the cytokines IL-12 and IL-10. Results are an average of triplicate wells ±SEM from representative experiment. (B) Supernatant nitrite measured by Greiss assay (Promega) and arginase enzyme activity from cell lysates at 12 hr polarization as described [1].

1. Wynn TA, Barron L, Thompson RW, Madala SK, Wilson MS, et al. (2011) Quantitative assessment of macrophage functions in repair and fibrosis. Current protocols in immunology / edited by John E.Coligan ...[et al.] Chapter 14.

**
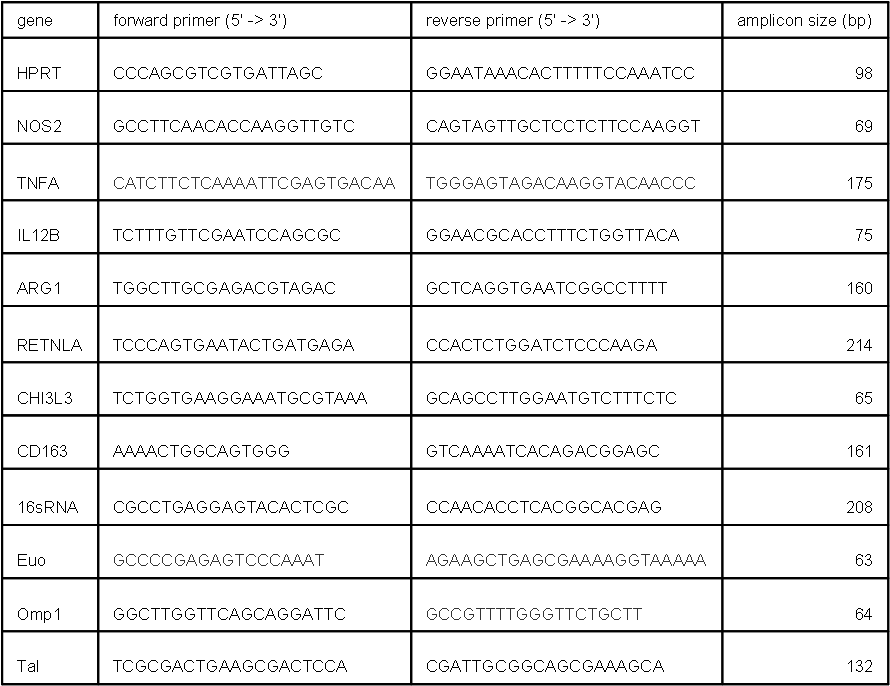
**

**Figure S2: Primer list.** Design and quality control discussed in methods.
